# Die Kasai-Hepatoportoenterostomie zur Behandlung der Gallengangatresie – Worauf kommt es an?

**DOI:** 10.1007/s00104-025-02259-2

**Published:** 2025-02-27

**Authors:** Omid Madadi-Sanjani, Uta Herden, Marie Uecker

**Affiliations:** 1 Klinik für Viszerale Transplantationschirurgie, Universitätsklinikum Hamburg-Eppendorf, Martinistraße 52, 20246 Hamburg, Deutschland; 2 Klinik für Kinderchirurgie, Medizinische Hochschule Hannover, Hannover, Deutschland

**Keywords:** Gallengangatresie, Leberzirrhose, Kasai, Lebertransplantation, Zentralisation, Biliary atresia, Liver cirrhosis, Kasai, Liver transplantation, Centralization

## Abstract

Die Gallengangatresie (biliäre Atresie, BA) ist ein seltenes Krankheitsbild des Neugeborenen unklarer Ätiologie. Die BA definiert sich über das Ausmaß der extra- und intrahepatischen Gallenwegsdestruktion, die innerhalb der ersten Lebensjahre zum Bild der Leberzirrhose führt. Die Lebertransplantation (LT) ist die einzige kurative Therapie der BA, einhergehend mit LT-assoziierten Risiken und Komplikationen. Doch auch über 60 Jahre nach ihrer Erstbeschreibung hat die Kasai-Hepatoportoenterostomie (KPE) einen wichtigen Stellenwert in der sequenziellen Behandlung der BA als primäre chirurgische Therapieoption, die ein längeres Überleben mit eigener Leber ermöglichen kann. Wir beleuchten chirurgische Schlüsselschritte der KPE und diskutieren relevante Aspekte.

## Abstract (en)

Biliary atresia (BA) is a rare disease in neonates of unknown etiology. BA is defined by the extent of extra- and intrahepatic bile duct destruction, which results in liver deterioration and cirrhosis within the first years of life. Liver transplantation (LT) is the only curative treatment for BA, accompanied by LT-associated risks and complications; however, more than 60 years after it’s first report, the Kasai hepatoportoenterostomy (KPE) is still an essential procedure in the sequential management of BA, as the primary surgical treatment option that can achieve long-term survival with a native liver. We highlight the key surgical steps of KPE and discuss relevant aspects.

## Hintergrund

Die Gallengangatresie (syn. biliäre Atresie [BA]) ist eine desaströse chirurgische Erkrankung des Neugeborenen [40]. Es wird vermutet, dass ein bisher unbekannter (pränataler) Trigger zu einer unkontrollierten inflammatorischen Destruktion der extra- und intrahepatischen Gallenwege führt, sodass die betroffenen Kinder innerhalb der ersten Lebensmonate ohne Behandlung eine Leberzirrhose entwickeln [29]. Die BA ist äußerst selten, mit einer Inzidenz von knapp 1:20.000 in Westeuropa, stellt aber global die häufigste Indikation für eine Lebertransplantation (LT) im Kindesalter dar [1]. Die BA wird deshalb unweigerlich mit der LT assoziiert, da es sich um die einzige kurative Therapieoption handelt. Doch das Behandlungskonzept beinhaltet eine weitere Komponente, einen palliativen chirurgischen Eingriff, der den inflammatorischen destruktiven Prozess nicht aufhält, jedoch eine biliäre Drainage herzustellen versucht, um die cholestatisch assoziierte Deterioration der Leber aufzuhalten oder zu verzögern [38].

Morio Kasai (1922–2008) und die BA sind unwiderruflich miteinander verbunden, denn auch knapp 70 Jahre nach der Erstbeschreibung der Behandlung eines 72 Tage alten Kindes mit einer Gallengangatresie wird die Prozedur als Kasai-Operation (syn. Kasai-Hepatoportoenterostomie [KPE]) bezeichnet [16, 31, 49]. Im nordjapanischen Sendai wurde 1955 die erste KPE durchgeführt, damals noch mittels Ableitung der Galle in das Duodenum [35]. Das Duodenum wurde in diesem Fall ungeöffnet auf die Porta hepatis genäht. Zum Erstaunen der Behandler hatte das Kind postoperativ gefärbte Stühle. Als das Kind trotzdem im weiteren Verlauf an Leberversagen verstarb, erfolgte die Autopsie durch Morio Kasai, der eine Fistel zwischen den intrahepatischen Gallenwegen und dem aufgenähten Duodenum feststellen konnte und daraus einen natürlichen Drang der Gallenwege zur Drainage konkludierte [35].

Mittlerweile ist die KPE der Goldstandard der Therapie bei Erstdiagnose einer BA im Neugeborenenalter, jedoch führt diese nur in knapp 50 % der Fälle zu einer temporären postoperativen biliären Drainage und nur 20–30 % der Patienten überleben nach der KPE mit ihrer eigenen Leber für einen längeren Zeitraum [2]. Das multimodale Konzept aus KPE, dem Management von Kindern mit einer Leberzirrhose und deren Komplikationen sowie der LT hat die Lebenserwartung der Patienten über die letzten Jahrzehnte exponentiell steigen lassen, sodass diese bei knapp 90 % für die ersten Lebensjahre liegt.

Doch auch wenn die KPE auf ihren palliativen Charakter und das Bridging bis zur LT reduziert wird, kann sie in den Händen spezialisierter Chirurgen auch ein langfristiges Überleben mit Eigenleber erreichen [8]. Die in *The Lancet* publizierte Arbeit von McKiernan et al. verglich die KPE-Ergebnisse von Zentren mit mehr (Gruppe A) und weniger (Gruppe B) als 5 KPE pro Jahr im Vereinigten Königreich (UK) und Irland der 1990er-Jahre [30]. Während das 5‑Jahres-Überleben mit Eigenleber in Gruppe A bei 61 % lag, konnte dieses Ergebnis im Vergleich nur bei 14 % der Kinder in Gruppe-B-Zentren erreicht werden. Das Gesamtüberleben lag in Gruppe-A- bei 91 % und in Gruppe-B-Zentren bei 75 % [30]. Auf Grundlage dieser Ergebnisse erfolgte in Großbritannien eine Zentralisierung der BA-Versorgung, mit letztlich drei verbliebenen Zentren, die weiterhin Patienten mit BA versorgen und die KPE durchführen dürfen [9, 11]. Wie wichtig dieser Schritt war, zeigt eine aktuelle Arbeit in *Annals of Surgery* von Davenport et al., in der die Outcomes von 867 Kindern mit BA, die zwischen 1999 und 2019 in UK (in den drei Zentren) behandelt wurden, analysiert werden [10]. In dieser Kohorte konnten 5‑ und 10-Jahres-Überlebensraten mit Eigenleber von 51 und 47 % erreicht werden und das 5‑ und 10-Jahres-Gesamtüberleben lag bei 92 und 91 %.

Doch erreichen wir in Deutschland annähernd vergleichbare Ergebnisse? Wir können uns hierbei auf eine kürzlich erschienene Arbeit berufen, die KPE-Ergebnisse der Jahre 2010 bis 2014 in Deutschland analysiert hat [26]. In diesem Zeitraum wurden insgesamt 160 KPE in 21 Zentren durchgeführt. Während das Überleben mit Eigenleber 2 Jahre nach KPE 29 % erreichte, lag das Gesamtüberleben bei 88 %. Somit waren die KPE-Ergebnisse deutlich schlechter, als die der zentralisierten Versorgung in Großbritannien (Tab. 1; [10, 26]). Auch diese Arbeit warf Diskussionen auf, die vor allem in den Fachgesellschaften geführt wurden. Im Jahr 2020 resultierte dies in einer deutlichen Empfehlung der Deutschen Gesellschaft für Kinderchirurgie (DGKCH), die KPE nur noch in ausgewiesenen Zentren durchzuführen, die in diesem Rahmen auch definiert wurden. Ob die Zentralisierung auch in Deutschland positive Effekte auf das Patientenoutcome haben wird, wird sich voraussichtlich erst in 5 bis 10 Jahren zeigen. Doch was macht die KPE genau aus und welche verschiedenen Schritte sind entscheidend?

**Tab. 1:** Ergebnisse der Kasai-Operation in europäischen Nationen mit Daten zum Gesamtüberleben sowie dem (transplantationsfreien) Überleben mit Eigenleber

Autor	Land	Zeitspanne	Patientenzahl	Gesamtüberleben	Überleben mit Eigenleber
Davenport et al. [9]	England und Wales	1999–2002	148	89 % (4J^a^)	51 % (4J)
Davenport et al. [11]	England und Wales	1999–2009	443	90 % (5J)	46 % (5J)
Davenport et al. [10]	England und Wales	1999–2019	867	92 % (5J) und 91 % (10J)	51 % (5J) und 47 % (10J)
Fanna et al. [14]	Frankreich	1986–2015	1428	92 % (1J), 82 % (5J), 80 % (10J), 78 % (20J), 76 % (30J)	41 % (5J), 35 % (10J), 26 % (20J), 22 % (30J)
Pakarinen et al. [37]	Nordisches Konsortium	2005–2016	158	88 % (5J)	53 % (5J)
Hukkinen et al. [19]	Finnland	2005–2016	36	95 % (2J)	78 % (2J)
De Vries et al. [48]	Niederlande	1987–2008	214	73 % (4J)	46 % (4J)
Wildhaber et al. [50]	Schweiz	1994–2004	48	92 % (2J)	43 % (2J)
Leonhardt et al. [23]	Deutschland	2001–2005	183	83 % (2J)	20 % (2J)
Madadi-Sanjani et al. [26]	Deutschland	2010–2014	173	88 % (2J)	29 % (2J)

^a^Die genannten Follow-up-Jahre

## Kasai-Hepatoportoenterostomie

### Cholangiographie

Die gesamte präoperative Diagnostik der BA beruht auf der Identifikation bzw. dem Ausschluss von Differenzialdiagnosen. Es gibt kein nichtinvasives diagnostisches Instrument mit einer ausreichenden Sensitivität zur zuverlässigen Diagnose der BA [13, 45]. Laborchemische Profile, Virusserologien, Genetik, die Echokardiographie, der Ultraschall sowie auch alternative und nicht überall verfügbare Bildgebungen wie die hepatobiliäre Sequenzszintigraphie (HBSS) können eingesetzt werden [3, 17, 20]. In der Regel bedarf es jedoch bei Ausschluss aller weiteren Diagnosen einer Darstellung der extra- und intrahepatischen Gallenwege.

Bereits Anfang der 2000er-Jahre gewann die endoskopisch retrograde Cholangiopankreatographie (ERCP) an Bedeutung, da eine Sensitivität von über 95 % (positiv prädiktiver Wert 92 %; negativ prädiktiver Wert 97 %) erreicht werden konnte [32, 41]. Eine europäische Umfrage zeigte jedoch die Probleme der technischen Umsetzung auf: Es handelt sich um eine in dieser Altersklasse selten durchgeführte Prozedur, mit ca. 5 ERCPs pro Jahr in den spezialisierten Zentren, und die Zahl von Zentren mit geeignetem und funktionierendem Equipment scheint in den letzten 10 Jahren tendenziell abzunehmen [21].

**Abb. 1:**
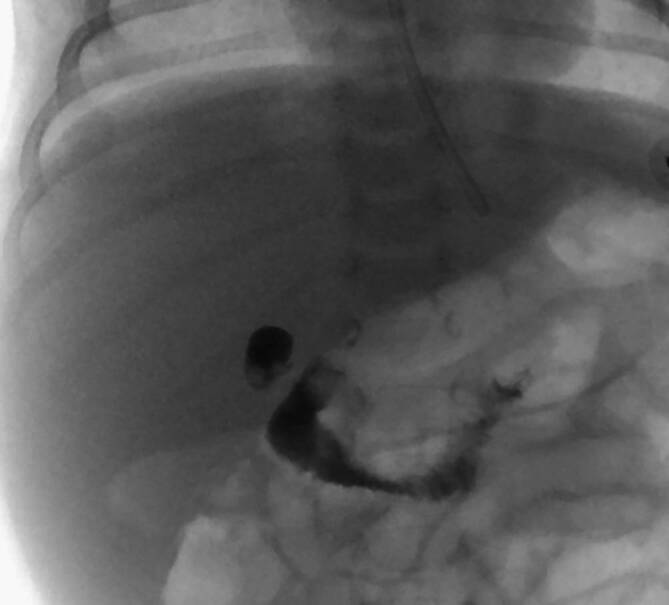
Das Bild einer intraoperativen Cholangiographie, mit Kontrastmittelgabe (Imeron 250 1:1 verdünnt) über die Gallenblase, mit Drainage nach intestinal, jedoch nicht zu identifizierenden intrahepatischen Gallenwegen (Ohi Typ II)

**Abb. 2:**
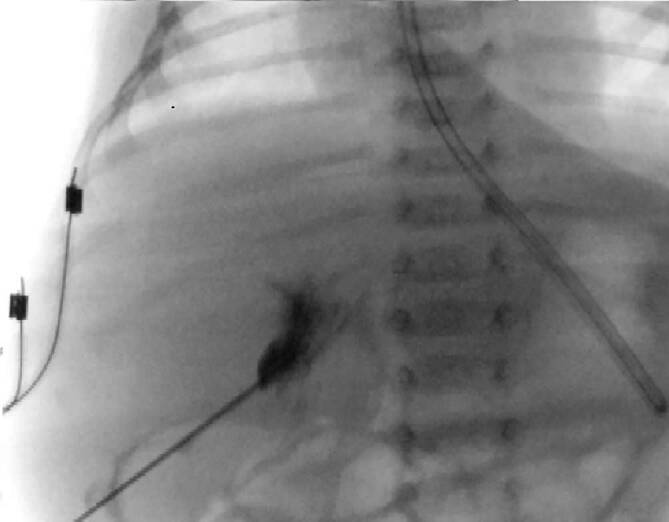
Intraoperative Cholangiographie (Imeron 250 1:1 verdünnt) über die hypoplastische Gallenblase, wobei sich kein Abfluss in die extra- und intrahepatischen Gallenwege darstellen lässt (Ohi Typ III)

Während die ERCP eine BA zumindest ausschließen kann, stellt sich für den Chirurgen jedoch die Frage, ob eine ERCP beim Vorliegen einer BA die intraoperative Cholangiographie obsolet werden lässt. Um diese Frage zu beantworten, muss zunächst klargestellt werden, dass die BA an sich ein heterogenes Krankheitsbild ist, bei der das Ausmaß der extrahepatischen Destruktion der Gallenwege hochvariabel ist. Deshalb sind Klassifikationen der BA im akademischen Gebrauch sehr entscheidend, da diese auch Informationen über die Prognose geben können. Neben einer französischen Klassifikation der BA, findet die Ohi-Klassifikation (Typ I–III) aus Japan regelhafte Anwendung [24]. Es kann hierbei in verschiedenem Maße eine Obstruktion des distalen Ductus hepaticus communis (DHC) vorliegen (Typ I), es können DHC-Obstruktionen mit patentem rechtem und linkem Gallengang vorliegen (Typ II; Abb. 1) sowie eine vollständige Destruktion des extrahepatischen Gallenwegssystem (Typ III; Abb. 2). In der klinischen Routine wird die BA häufig auf den Typ III nach Ohi reduziert. Die amerikanische Arbeitsgruppe um Superina et al. konnte in einer Kohorte von 244 Patienten zeigen, dass diese Subgruppen sehr wohl einen Einfluss auf das Überleben mit Eigenleber haben und signifikant schlechtere Outcomes für Betroffene mit einer Ohi-Typ-II- und -III-BA zu erwarten sind [44].

Das Ausmaß der extrahepatischen Gallenwegsdestruktion sowie der Ausbildung intrahepatischer Gallenwege kann im Falle einer bei der BA typischerweise vorliegenden Obstruktion nicht mit der ERCP identifiziert werden – somit sollte eine Cholangiographie bei betroffenen Patienten stets erfolgen.

Dies kann mittels transhepatischer Punktion erfolgen, um Spillage des Kontrastmittels zu verhindern, wie es z. B. durch die interventionelle Radiologie des Sick Children’s Hospital in Toronto praktiziert wird, wo dieser Eingriff als präoperative perkutane transhepatische Cholezystocholangiographie durchgeführt wird [36, 39]. Bei der traditionellen operativen Cholangiographie wird die Gallenblase entweder mit einer feinen Nadel punktiert oder partiell zum Einbringen einer Kanüle eröffnet. Über diese Zugangswege kann Kontrastmittel simultan zur radiographischen Darstellung appliziert werden. Wichtig ist, dass die Cholangiographie nicht mit dem Abfluss des Kontrastmittels in das Duodenum beendet wird, da diese Konstellation auch im Rahmen der Ohi-Typ-II-BA vorliegen kann. Hieraus ergibt sich der Vorteil der intraoperativen Cholangiographie, dass der Abfluss nach distal zuverlässig mittels Pringel-Manöver oder isoliertem manuellem Verschluss des Gallenwegs vor dem Duodenum kurzzeitig gestoppt wird, um die proximalen biliären Strukturen sicher darstellen zu können. Nur eine eindeutige Darstellung der intrahepatischen Gallenwege mit sichtbarer Aufzweigung („Gallenwegsbäumchen“) sowie des Abflusses in das Intestinum ist ein sicherer BA-Ausschluss.

### Exploration

Wenn die Diagnose der BA bestätigt ist, sollte bei fast allen Patienten eine KPE erfolgen. Es gibt nur seltene Ausnahmen, wie z. B. ein bereits grobknotiger zirrhotischer Umbau der Leber mit ausgeprägtem Aszites, bei denen entschieden werden kann, eine primäre Lebertransplantation (pLT) durchzuführen [12]. In den großen europäischen Zentren macht die pLT nur weniger als 5 % aller Fälle aus und Daten aus Großbritannien, Frankreich und auch Deutschland zeigen, dass selbst Patienten im fortgeschrittenen Alter von einer KPE als Bridging zur LT profitieren können, während eine kleine Kohorte dieser Patienten sogar ein langfristiges Überleben mit Eigenleber erreichen kann [6, 15, 46].

**Abb. 3:**
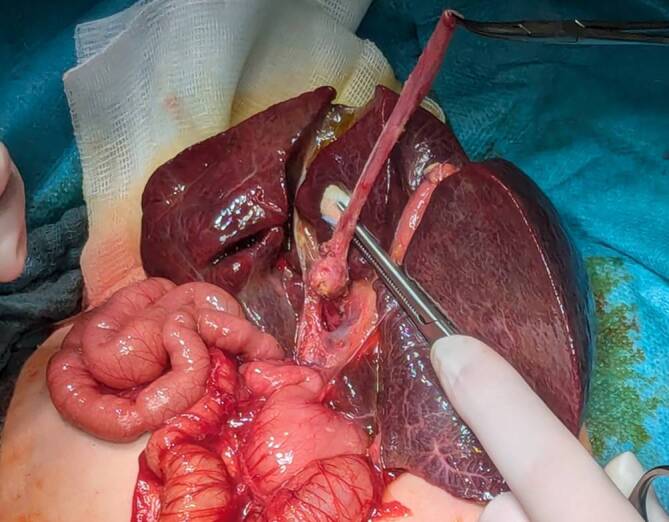
Nach Durchtrennung des Lig. falciforme und triangulare sinistra kann die Leber vollständig über das Hautniveau luxiert werden. Hier zeigt sich eine zystische Struktur am Hilus ohne Abfluss nach distal, jedoch mit Nachweis hypoplastischer intrahepatischer Gallenwege (Ohi Typ I)

**Abb. 4:**
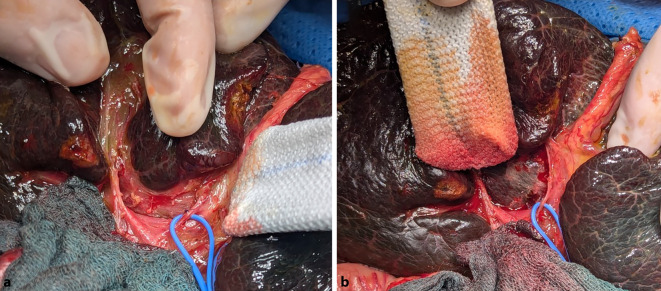
**a** Freigelegte Porta hepatis bei einer Gallengangatresie (biliäre Atresie) Typ III nach Ohi, mit Darstellung der fibrotischen „Plattenregion“ und ohne Nachweis extrahepatischer Gallenwege; **b** die Porta hepatis nach scharfer Exzision der fibrotischen Platte

Die KPE erfolgt in der Regel über eine quere Oberbauchlaparotomie. Ein Großteil der KPE-Zentren, wie z. B. das King’s College London, praktizieren eine ausgeprägte Lebermobilisation mit Durchtrennung des Ligamentum falciforme sowie triangulare sinistra, um die Leber daraufhin über das Hautniveau zu luxieren (Abb. 3 und 4a). Dieser Eingriff vereinfacht die Exposition der Porta hepatis und macht eine radikalere Exzision entlang der Pfortader möglich (Abb. 4b). Diese Lebermobilisation bedarf insbesondere einer kinderanästhesiologischen Expertise, da das Abknicken der Lebervenen zu temporären kardialen Funktionseinschränkungen führen kann, was den Patienten hämodynamisch kompromittiert und entsprechende Maßnahmen (Flüssigkeitssubstitution, Katecholamine) erforderlich macht. Da die Lebermobilisation während der KPE aufgrund entstehender Adhäsionen mit einer erschwerten Hepatektomie während der potenziell nachfolgenden LT assoziiert wird, ohne dass dies jemals systematisch im Rahmen einer Studie aufgearbeitet wurde, verzichten einige Zentren auf die Mobilisation, belassen die Leber orthotop in situ und setzen Rahmensysteme zur besseren Exposition ein (z. B. Thomson-Retractor).

### Präparation der Porta hepatis

**Abb. 5:**
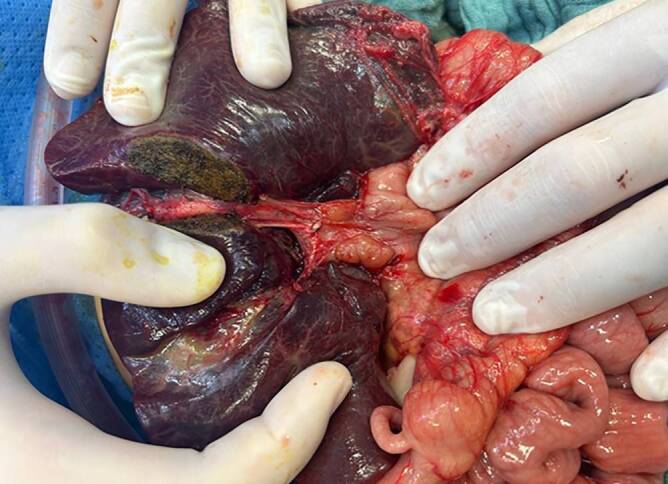
Der Recessus Rex wurde für eine bessere Exposition des linken Pfortaderstammes durchtrennt. Die fibrotische Platte ist bereits exzidiert

Auch wenn früher die vaskulären Strukturen des Ligamentum hepatoduodenale einzeln dargestellt und angeschlungen wurden, wird in den meisten Fällen hierauf verzichtet, um Adhäsionen in diesem Bereich für die potenziell nachfolgende LT zu reduzieren. Trotzdem müssen die rechte und linke Leberarterie sowie in besonderen Fällen aberrante Arterien identifiziert und geschützt werden. Die Pfortadergabel muss sicher dargestellt werden, da sich die Narbenplatte der Porta hepatis bei der BA in diesem Bereich einbettet. Hierbei kann es notwendig werden, einzelne Pfortaderäste abzutragen, um eine möglichst große Fläche für die Resektion und nachfolgende Ableitung zu schaffen. Es kann hierbei je nach Länge und Breite des Recessus Rex notwendig werden, diesen zu eröffnen, um insbesondere entlang der linken Pfortader größere Flächen der Narbenplatte resezieren zu können (Abb. 5). Das Abtragen der Narbenplatte muss scharf erfolgen, mit der Schere oder mit einem Skalpell und ohne den Einsatz von Koagulation, da die Sorge besteht hierdurch kleinste Gallenwege zu obliterieren. Resektionen in das Leberparenchym hinein sollten nicht erfolgen, da dies zu Narbenbildungen oder Blutungen führen kann, welche wiederum feinste Gallenwege verschließen können.

### Intestinale Anastomose

Der Anschluss der Porta hepatis an das Intestinum erfolgt als Y‑Roux-Rekonstruktion. Es sollte die Flexura duodenojejunalis aufgesucht und mindestens 20 cm distal hiervon das Jejunum durchtrennt werden. Hier kann nun die Fußpunktanastomose angelegt werden, als Jejunojejunostomie. Dies kann als End-zu-Seit- oder als iso- und anisoperistaltische Seit-zu-Seit-Anastomose erfolgen. Der Mindestabstand zu Treitz sollte unbedingt eingehalten werden, da es im Rahmen der LT zu einer Revision der Fußpunktanastomose kommen kann und ausreichend Mobilität der Schlinge vorliegen sollte.

Von äußerster Wichtigkeit ist auch die Länge der retrokolisch an die Porta hepatis geführten Roux-Schlinge. Diese sollte mindestens 40 cm lang sein, da im Rahmen der potenziell folgenden LT diese gekürzt werden muss und deshalb eine ausreichende Länge vorliegen sollte. Es ist auch hier zu bedenken, dass dieser Vorgang bei der Notwendigkeit von Retransplantationen wiederholt werden muss. Die Länge der Y‑Roux-Schlinge nach LT soll zudem protektiv gegen aszendierende Cholangitiden wirken. Das retrokolische Fenster für die Roux-Schlinge sollte im besten Fall nicht zu zentral im Mesenterium des Colon transversum gewählt werden, da ein laterales Durchführen im Bereich der rechten Kolonflexur eine bessere Positionierung der Schlinge im Rahmen einer LT mit einem linkslateralen Split ermöglicht.

Eine Ausnahme stellt die syndromale Form der BA dar, die häufig mit einer Malrotation einhergeht. Hierbei sollte unbedingt die Länge der Roux-Schlinge beibehalten werden (40–45 cm), diese wird jedoch direkt (antekolisch) auf die Porta hepatis platziert.

Alle mesenterialen Lücken müssen sicher verschlossen werden, da es zu inneren Hernien mit Obstruktionen und einem mechanischen Ileus kommen kann.

### Hepatoportoenterostomie

**Abb. 6:**
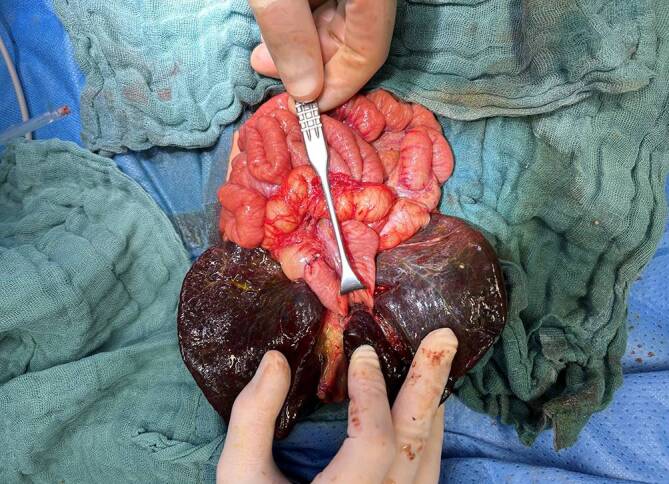
Die retrokolisch an die Porta hepatis positionierte Y‑Roux-Schlinge (Länge 45 cm) wurde mit PDS-6/0-Einzelknopfnähten an das Leberparenchym (Hepatoportoenterostomie) fixiert

Für die Hepatoportoenterostomie wird die abgesetzte Jejunalschlinge in der Regel antemesenterial eröffnet. Die Anastomose wird dann mittels Einzelknopfnähten, häufig mit monophilem Nahtmaterial (z. B. PDS 6/0) angelegt (Abb. 6). Um die Ablauffläche so großflächig wie möglich zu gestalten, kann für die Hinterwand eine seromuskuläre Adaption des Jejunums an der Adventitia der vaskulären Strukturen erfolgen.

Drainagen müssen nicht zwingend platziert werden, können jedoch bei den zu erwartenden größeren Aszitesvolumina postoperativ als Indikator dienen und vor allem den abdominellen Druck, z. B. auf die Pfortader oder auch auf die Anastomosenfläche, reduzieren. Fördermengen müssen dann engmaschig bilanziert und mittels Humanalbumin (teilweise) ersetzt werden. Bei zu langer Liegedauer der Drainagen entwickeln sich Fistelkanäle, die bei einigen Kindern persistieren können.

### Stellenwert der Laparoskopie

Die minimal-invasive KPE wird teils kontrovers diskutiert, da sie in einigen Regionen Südostasiens bereits zur Standardversorgung gehört, global jedoch nicht an die erfolgreichen Ergebnisse aus Japan angeknüpft werden konnte [43]. Die einzige prospektive Studie hierzu von Ure et al. musste frühzeitig abgebrochen werde, da die Rate frühzeitiger LT in der Gruppe laparoskopischer KPE signifikant höher war als in der konventionellen (offenen) KPE-Kohorte [47]. Die Fortführung der Studie war für das Studienteam deshalb ethisch nicht vertretbar. Eine Folgeanalyse der gleichen Gruppe zeigte zudem, dass die Laparoskopie keine Vorteile für die nachfolgende LT beinhaltete [34]. Operationsdauer der LT, der intraoperative Blutverlust sowie auch die Reoperationsrate waren für beide KPE-Gruppen gleichwertig. Die Arbeitsgruppe um Chan et al. konnte zudem zeigen, dass die erneute Umstellung von der laparoskopischen auf die offene, konventionelle KPE mit einer signifikanten Verbesserung des Outcomes einherging, sodass die Großzahl der internationalen KPE-Zentren aktuell die offene KPE bevorzugt [4].

## Diskussion

Das Management der Gallengangatresie (syn. biliäre Atresie [BA]) kann zu verschiedenen Zeitpunkten entscheidend beeinflusst werden. Das Alter zum Zeitpunkt der Diagnose sowie der Kasai-Hepatoportoenterostomie (KPE) sind wichtige prognostischen Faktoren [33]. Die KPE innerhalb der ersten 60 Lebenstage ist mit einem signifikant besseren transplantatfreien Überleben (mit Eigenleber) assoziiert, weshalb der Eingriff so früh wie möglich erfolgen sollte. In einer neuerlichen europäischen Umfrage liegt das durchschnittliche KPE-Alter jedoch bei knapp über 60 Lebenstagen, weshalb Früherkennungsmaßnahmen diskutiert werden [22]. Auf Initiative zahlreicher pädiatrischer Leberzentren in Deutschland wurde im November 2023 gemäß dem Entscheid des Gemeinsamen Bundesausschusses (G-BA) eine Stuhlfarbenskala in das Vorsorgeheft von Neugeborenen integriert, um Eltern dabei zu helfen, acholische Stühle und damit lebensgefährliche Hepatopathien wie die BA frühzeitig zu erkennen [25, 28]. Erfahrungen aus Japan und Taiwan mit solch einem Screening konnten bereits ein signifikant besseres transplantatfreies Überleben der Betroffenen bestätigen [7, 18].

Die Diagnostik und Therapie der BA sollten zwingend in spezialisierten Zentren erfolgen, da auch dies nachweislich einen Effekt auf das Gesamt- sowie auch transplantatfreie Überleben hat [11, 19]. Die offene KPE mit intraoperativer Cholangiographie stellt nach wie vor den Goldstandard der Primärdiagnostik und -therapie von BA-Patienten dar. Zahlreiche beschriebene Modifikationen der Kasai-Operation in den letzten Jahrzehnten konnten sich international nicht durchsetzen [42]. Stomata der Roux-Schlinge oder antirefluxive Prozeduren zur Reduktion der postoperativen Cholangitis sowie auch beschriebene extensive Präparationen mit teils Leberresektionen konnten das Überleben mit Eigenleber nicht nachweislich verbessern, sodass die KPE nach wie vor weitestgehend unverändert im Vergleich zur Originalbeschreibung durchgeführt wird.

Die makroskopische Lebermorphologie, anatomische Variationen sowie die Rekonstruktionen mit Maßangaben (insbesondere Angaben zum Verlauf und der Länge der Y‑Roux-Schlinge) müssen zwingend detailliert angegeben werden, um auch die potenzielle Transplantation besser zu planen. Um das Outcome der KPE langfristig und zuverlässig zu verbessern, sollte neben der bereits laufenden Zentralisierung deshalb eine weitestmögliche Standardisierung des Eingriffes angestrebt werden, mit dem Ziel zumindest europäische Versorgungsergebnisse, wie aus Großbritannien oder Frankreich, zu erreichen [5, 10, 14].

Zahlreiche adjuvante Behandlungen, wie der Einsatz postoperativer Steroide, werden kontrovers diskutiert, da sich in den bisherigen Studien inkonklusive Ergebnisse zeigen und somit keine uneingeschränkte Empfehlung erfolgen kann [27].

Im Rahmen der DGKCH-Initiative zur Zentralisierung der BA-Versorgung in Deutschland galt der Aufruf, dass die Zentren ihre KPE-Ergebnisse transparent offenlegen müssen. Für die Dokumentation der Ergebnisse steht hierfür das EBAR (European Biliary Atresia Registry) der Europäischen Referenznetzwerke für seltene Lebererkrankungen (ERN RARE-LIVER) zu Verfügung, welches neben der Analyse der deutschen KPE-Ergebnisse auch einen Vergleich mit weiteren europäischen Zentren zulässt und somit für eine Bewertung der deutschen BA-Versorgung erforderlich ist. Diese Auswertungen müssen in Zukunft die Versorgungslandschaft verbessern und es müssen klare Benchmarks definiert werden.

Letztlich wird in der Zusammenarbeit der pädiatrischen Leberzentren der Schlüssel zur Versorgungsverbesserung liegen, mit dem gemeinsamen Ziel möglichst vielen Kindern ein (langfristiges) Leben mit ihrer eigenen Leber zu ermöglichen.
